# The influence mechanism of OBE-CDIO integration on engineering literacy: an empirical study based on student perceptions

**DOI:** 10.3389/fpsyg.2026.1812059

**Published:** 2026-05-29

**Authors:** Qi Yan, Licheng Li, Renfei Liu, Yiliu Pu, Peng Cui

**Affiliations:** 1 Nanjing Forestry University, Nanjing, China; 2 Umea University, Umea, Sweden

**Keywords:** cognitive-behavioral mechanism, educational environment quality, engineering literacy development, OBE-CDIO integrated education, structural equation modeling

## Abstract

**Introduction:**

Engineering education is shifting from knowledge transmission to competence construction. The integration of Outcome-Based Education (OBE) and Conceive-Design-Implement-Operate (CDIO) represents this direction, yet research lacks empirical examination of the complete student perception-psychological process-competence chain. This study constructs an integrated model to explore how students’ perceived OBE-CDIO environment quality influences engineering literacy development through its multidimensional structure.

**Methods:**

A cross-sectional research design was employed, administering a retrospective questionnaire survey to 366 graduates from a Double First-Class university in China who had experienced the OBE-CDIO integrated curriculum. Structural Equation Modeling (SEM) was used for higher-order factor analysis and path testing. Core constructs included: Integrated Education Environment Quality (a second-order latent variable measured by Curriculum Objective Alignment X1, Practical Project Richness X2, and Perception of Dual-Qualification Faculty Ratio X3) and Integrated Engineering Literacy (a higher-order latent variable comprising two first-order factors: Cognitive Internalization Literacy and Practical Externalization Literacy).

**Results:**

The measurement model demonstrated excellent fit (χ^2^/df = 2.41, CFI = 0.962, TLI = 0.957, RMSEA = 0.062). The higher-order structural equation model revealed a strong total effect of integrated education environment quality on integrated engineering literacy (*β* = 0.72, *p* < 0.001). Mediation path analysis uncovered a key mechanism: environmental quality significantly enhanced students’ cognitive internalization literacy (*β* = 0.65), which in turn strongly drove the development of their practical externalization literacy (*β* = 0.80). Among the three dimensions of environmental quality, curriculum objective alignment (*β* = 0.282) contributed the most, followed by practical project richness (*β* = 0.222) and perception of dual-qualification faculty ratio (*β* = 0.170). In the sequential mediation model (Model B), total effect decomposition revealed that curriculum objective alignment had the largest total effect on practical externalization literacy (0.458), followed by practical project richness (0.426) and dual-qualification faculty ratio (0.395).

**Conclusion:**

OBE-CDIO integration constructs an empowering environment where clear objectives promote knowledge internalization, which transforms into practical competencies under project and faculty guidance. This provides an empirically-based, stage-specific mechanism map for student-centered and outcome-oriented engineering education reform. The study offers a student-centered psychological perspective, bridging the gap between macro-level educational design and micro-level competence development in engineering education.

## 1 Introduction

Driven by the Fourth Industrial Revolution and international engineering education accreditation (Becker and Woessmann, 2024), global engineering education (Cai and Hu, 2025) is undergoing a profound paradigm revolution. The core of this revolution is shifting from focusing on “what the teacher taught” to “what the student can do.” Outcome-Based Education (OBE), as an advanced educational philosophy, provides a clear logical starting point for this revolution — designing the curriculum system backwards from peak outcomes (Liu et al., 2023). However, OBE itself does not specify the concrete path to achieve these outcomes. The Conceive-Design-Implement-Operate (CDIO) model (Guan et al., 2023), with its highly contextualized, integrative, and practical characteristics (Jiawen et al., 2024), serves as an ideal vehicle for cultivating complex engineering competencies. The integration of the two is considered capable of ensuring both the directionality and practicality of education.

Nevertheless, current research on OBE-CDIO integration exhibits two distinct disconnects. First, there is an environment-individual disconnect. Numerous studies describe how courses are designed and projects are implemented, yet few systematically evaluate, from the student perception perspective, the impact of this integrated environment as a whole on their learning experience (Yuan et al., 2026). Students are the ultimate users of education; their psychological representation of the educational environment (e.g., whether they perceive goals as clear, practice as substantial, and teachers as credible) is the logical starting point for any educational intervention to take effect (Noushad, 2024). Second, there is an input–output mechanism disconnect. Most research stops at proving that the integrated model can improve overall performance or satisfaction, but how it improves — the specific psychological and behavioral mechanisms influencing different dimensions and levels of competence — remains a black box (Hariharasakthisudhan et al., 2025). Engineering literacy is a multi-level, multidimensional complex construct, encompassing levels from knowledge comprehension to complex problem-solving, and from personal skills to teamwork and social responsibility. An effective educational environment likely exerts differential, even sequential, influence paths on different levels of ability.

To bridge these disconnects, this study introduces perspectives from educational psychology and advanced statistical modeling. We conceptualize students’ perceptions of curriculum objectives, practical projects, and faculty characteristics (Ma et al., 2024) as a unified higher-order psychological construct of Empowering Educational Environment Quality. Simultaneously, drawing on Bloom’s Taxonomy of Educational Objectives and engineering accreditation standards, we deconstruct graduation requirements into two interrelated yet distinct competence dimensions: Cognitive Internalization (e.g., knowledge, analysis, lifelong learning) and Practical Externalization (e.g., design, communication, project management, social responsibility) (Benita and Matos, 2021). Based on social cognitive theory and the Input-Process-Output (IPO) model (Gutiérrez-Aguilar et al., 2024), we propose a sequential competence development hypothesis: a high-quality integrated education environment will first powerfully promote students’ deep cognitive internalization of engineering knowledge (Lee and Tseng, 2024); this solid cognitive internalization will then serve as the cognitive fuel and confidence cornerstone for effective action in explicit, complex engineering practice and social contexts, thereby driving excellent performance in practical externalization competencies.

Therefore, this study aims to empirically test the following core questions through a higher-order structural equation model: (1) How strong is the impact of the OBE-CDIO integrated education environment as a whole on students’ overall engineering literacy? (2) Are there differences in the paths through which this environment influences the two types of competencies: cognitive internalization and practical externalization? (3) Does a sequential mediation path exist from cognitive internalization to practical externalization? The findings will not only provide robust evidence based on student perceptions for the effectiveness of OBE-CDIO integration but also map its psychological mechanism map for promoting student competence development, offering precise theoretical guidance and practical strategies for deepening engineering education reform.

## 2 Literature review and theoretical framework

### 2.1 From dispersed features to holistic environment: the psychological connotation of educational environment quality

The educational environment extends far beyond physical space; it is a field of meaning constituted by social, pedagogical, and psychological factors. From the student perception perspective, the core characteristics of the OBE-CDIO integrated environment can be summarized as three points: goal clarity, practical challenge, and instructional support. Goal clarity (derived from OBE) reduces learning uncertainty and cognitive load, providing students with a navigation system for self-regulation. Practical challenge (derived from CDIO) creates necessary cognitive conflict and skill application scenarios, serving as the training ground for competence development (Makel et al., 2015). Instructional support (especially from dual-qualification faculty — instructors who possess both academic credentials and industry-derived practical qualifications, such as professional certifications or sustained industry engagement) provides the scaffolding and role models needed for the transition from novice to proficient (Ho et al., 2024). These three aspects are not isolated but mutually reinforcing, jointly constituting an empowering learning ecosystem that is challenging yet supportive, directional, and practical. Grounded in Moos’s (1982) social-ecological framework of educational environments and Marsh’s (1990) hierarchical construct theory, the three dimensions of goal clarity, practical challenge, and instructional support are conceptualized as first-order indicators loading onto a broader higher-order latent construct, namely Educational Environment Quality. This higher-order specification is theoretically defensible, as the three dimensions represent distinct yet correlated features of the same underlying psychosocial climate. Integrating them into the higher-order construct of Educational Environment Quality aligns with the holistic view in psychology, enabling a more concise and powerful capture of the overall psychological effect of the environment on students (Ouyang, 2025). The empirical validation of this higher-order structure, including factor loadings and model fit indices, is presented in Section 4.1 (Measurement Model).

### 2.2 The multidimensional deconstruction of engineering literacy: cognitive internalization and practical externalization

The ultimate goal of engineering education is to cultivate talents with complete engineering literacy. Drawing on the revised Bloom’s taxonomy, we can distinguish engineering literacy into two closely related but distinct dimensions: Cognitive Internalization Literacy and Practical Externalization Literacy (Lim et al., 2024). This distinction is not intended to replace Bloom’s hierarchical categories but to aggregate them for the specific purpose of testing a sequential mediation hypothesis. The Cognitive Internalization dimension primarily draws on the remember, understand, analyze, and evaluate cognitive processes, reflecting depth of knowing. The Practical Externalization dimension draws on the apply and create processes, plus the socio-technical competencies (e.g., teamwork, communication, ethics) emphasized in engineering accreditation standards. This aggregation is analytically necessary for structural equation modeling of indirect effects and is consistent with established practices in educational psychology (Anderson et al., 2000; Pretorius, 2018). Cognitive Internalization Literacy focuses on the individual’s internal understanding, analysis, evaluation, and metacognitive management of engineering knowledge, principles, and methods (D’Souza et al., 2023). It corresponds to graduation requirements such as Engineering Knowledge, Problem Analysis, Research, Use of Modern Tools, and Lifelong Learning, with its core lying in the depth of knowing and thinking. Practical Externalization Literacy emphasizes the ability to comprehensively apply knowledge, engage in design creation (Song, 2024), collaborate with others, manage projects, and fulfill ethical responsibilities in authentic or simulated socio-technical contexts. It corresponds to Design/Development of Solutions, Individual and Teamwork, Communication, Project Management, Engineering and Society, Environment and Sustainability, and Professional Ethics (Yucheng and Jiong, 2024, with its core lying in the breadth of acting and taking responsibility. This distinction holds significant theoretical value, as it not only acknowledges the objective gap between knowing and doing but also implies that the transformation from the former to the latter may be a nonlinear process requiring specific educational conditions (Gao and Mohamad, 2025).

### 2.3 The dual-cycle OBE-CDIO integrated education model: a systematic theoretical framework

**Figure 1:**
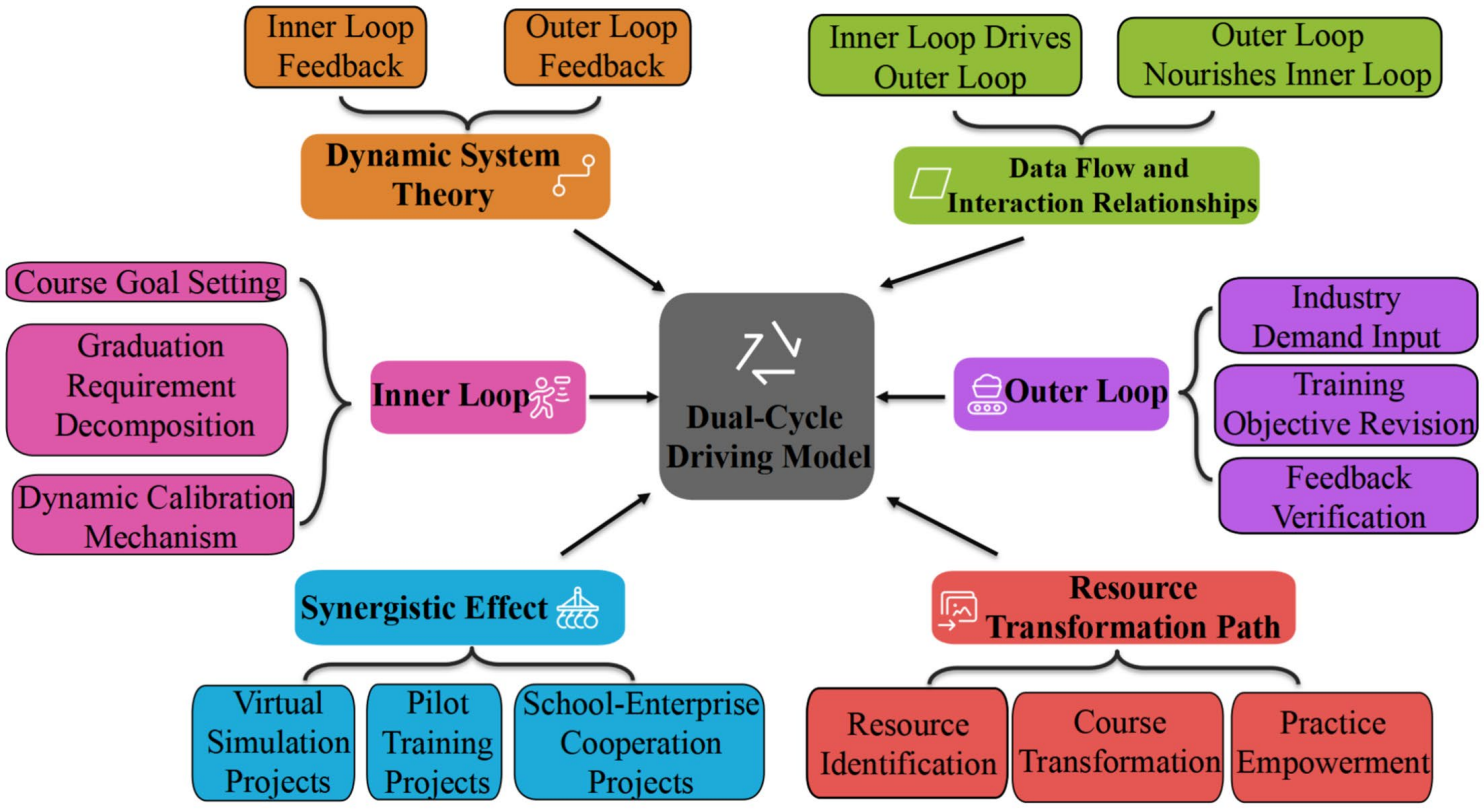
Theoretical framework of the dual-cycle OBE-CDIO integrated education model.

To build a bridge between macro-level educational design and micro-level competence development, we constructed the Dual-Cycle OBE-CDIO Integrated Education Model. Its theoretical framework is shown in Figure 1. This model consists of two nested, dynamically interacting cycles (Gomaa, 2023): the Inner Cycle (Objective Calibration Cycle) and the Outer Cycle (Demand Feedback Cycle).

The integration of OBE and CDIO in this study was necessitated by a specific curricular gap observed in the Chemical Engineering program at Nanjing Forestry University. OBE provided clear, measurable graduation outcomes and a philosophy of backward design, yet it did not specify concrete pedagogical pathways for students to achieve those outcomes. Conversely, CDIO offered rich, project-based learning environments that engaged students in authentic engineering tasks, but its implementation often lacked systematic alignment with terminal program outcomes. This dual limitation created variability in student attainment, prompting the formulation of an integrative vision that combines OBE’s directional clarity with CDIO’s practical engine.

The Dual-Cycle model presented in Figure 1 is a theoretical synthesis developed by the authors, grounded in three distinct sources. First, a systematic review of OBE implementation literature confirmed the recurrent challenge of translating outcome statements into actionable learning designs. Second, a review of CDIO literature revealed persistent difficulties in aligning project-based activities with program-level outcomes. Third, and most importantly, the model distills 4 years (2019–2023) of practical experience implementing OBE-CDIO integration in the Chemical Engineering program at Nanjing Forestry University, including iterative refinements based on graduate feedback and industry consultation. Thus, the model represents a practitioner-informed theoretical contribution, not a direct adoption from any single source.

The conceptual novelty of the Dual-Cycle model lies in explicitly distinguishing two functionally distinct but interacting loops, rather than treating continuous quality improvement as a single undifferentiated cycle. This distinction draws from Argyris and Schön’s (1978) organizational learning theory, which differentiates single-loop learning (correcting actions within existing frameworks) from double-loop learning (questioning and revising the frameworks themselves). In the present model, the Inner Cycle operationalizes single-loop learning by ensuring internal curricular coherence — mapping course objectives to program outcomes and adjusting instructional designs accordingly. The Outer Cycle operationalizes double-loop learning by capturing external industry feedback and feeding it back into program-level outcome revision, thereby reshaping the framework within which the Inner Cycle operates. This dual-loop specification advances beyond standard OBE implementations by explicitly theorizing the interaction between stability (curricular calibration) and adaptability (demand responsiveness), rather than treating them as a single, undifferentiated quality assurance process.

The Inner Cycle (Objective Calibration Cycle) is the core regulatory mechanism of the educational process, primarily functioning to ensure high consistency between course objectives and program learning outcomes (Nichalls-Spencer and Roofe, 2023). Guided by the OBE philosophy, it designs the curriculum system and learning objectives backwards from the pre-defined peak outcome (Graduation Requirements) (Du et al., 2023). For example, in the case of this study—the Forest-based Fine Chemicals specialization within the Chemical Engineering program at Nanjing Forestry University — the program aims to equip students with the technology for converting forest biomass resources and manufacturing fine chemical products. Within the Inner Cycle, specific learning objectives for each relevant course (e.g., Biomass Chemistry, Fine Organic Synthesis) must be meticulously calibrated based on this overall program objective, ensuring course content is closely aligned with program outcomes, allowing for the stepwise accumulation of student competencies (Alamon et al., 2024).

The Outer Cycle (Demand Feedback Cycle) focuses on the dynamic adaptation of the educational system to the external industrial environment (Wint and Nyamapfene, 2023). It emphasizes continuously capturing industry technology trends and talent demand changes through sustained university-industry collaboration, industry research, and graduate tracking, effectively feeding this information back into the revision and optimization of the cultivation plan (Campbell, 2023). For instance, when the market’s demand for the research and development of a certain type of new fine chemical products derived from forest resources intensifies (Hakamada et al., 2023), the Outer Cycle mechanism would prompt the university to timely adjust the curriculum by adding relevant theoretical modules or practical projects to cultivate students’ adaptability to new challenges.

The Inner and Outer Cycles do not operate in isolation but form a synergistic, evolving closed-loop system. This conceptualization is informed by systems theory (Newman and Newman, 2020), which emphasizes interdependence among system components, feedback loops as regulatory mechanisms, and the maintenance of dynamic equilibrium through continuous mutual adjustment. The objective calibration of the Inner Cycle provides a stable core and direction for education (corresponding to Goal Clarity within Educational Environment Quality), while the demand feedback of the Outer Cycle injects vitality and timeliness into the educational content (partly related to the industry linkage behind Practical Project Richness and Dual-Qualification Faculty) (Qadir et al., 2025). From a dynamic systems theory perspective (Piniel and Albert, 2025), the stability of the Inner Cycle and the adaptability of the Outer Cycle empower each other: a clear objective system provides a baseline and focus for adapting to external changes; continuous external feedback drives the Inner Cycle to make constant fine-tunings, keeping the educational system sensitive to industrial development. This Dual-Cycle model constitutes the operational logic of macro-level educational design (Guarda, 2025). The Empowering Environment → Cognitive Internalization → Practical Externalization pathway (Le Jeune et al., 2025), which is the focus of this study, is the micro-level competence development mechanism occurring at the individual student level within this macro framework. Together, they elucidate the complete action spectrum of OBE-CDIO integrated education from system design to individual development.

It is important to clarify that the empirical component of this study does not directly measure the dynamic, processual features of the Dual-Cycle model (e.g., frequency of curriculum revision meetings, intensity of industry feedback loops). Rather, the study operationalizes the perceived outputs of a well-functioning Dual-Cycle system as experienced by students. Specifically, effective Inner Cycle operation should result in students perceiving high curriculum objective alignment; effective Outer Cycle operation should result in students perceiving practical project richness and dual-qualification faculty support. Thus, the three measured environmental features serve as indicators of a mature Dual-Cycle system’s outcomes, not as direct measures of its internal processes.

### 2.4 Hypothesis development: a sequential competence development model

Integrating the above macro model and micro perspective, we propose the core theoretical framework of this study at the individual level: the Empowering Environment → Cognitive Internalization → Practical Externalization sequential influence model. This framework is informed by Bandura’s (1986) Social Cognitive Theory, which emphasizes triadic reciprocal causation among environment, person, and behavior. Social Cognitive Theory explains the psychological mechanism of learning but does not inherently structure the temporal sequence of learning events. To provide this temporal structure, the Input-Process-Output (IPO) model is adapted from organizational psychology (Llgen et al., 2005) and its subsequent applications in educational settings (Gozan et al., 2024). The logical convergence of the two theories lies in the mediating role of cognitive processes: the educational environment (Input) shapes students’ cognitive internalization (Process), which in turn drives the manifestation of practical competencies (Output). This combined framework has been empirically validated in recent educational psychology research (Brown et al., 2016). This model includes the following specific research hypotheses:

The sequential competence development hypothesis is formulated under three core assumptions: (a) temporal precedence — cognitive internalization (knowing) must temporally and logically precede practical externalization (doing); (b) mediation — the influence of the educational environment on complex practical performance is largely indirect, operating through cognitive processes; and (c) developmental hierarchy — competence development unfolds from foundational knowledge acquisition to applied action. This hypothesis directly challenges an alternative “direct-effects” account, which posits that environmental features such as practical projects and faculty support directly produce practical competencies without requiring deep cognitive mediation. This alternative is implicitly assumed in many descriptive OBE-CDIO studies but has rarely been tested. Thus, our hypothesis strives to empirically adjudicate between these competing accounts.

*H1* (Environment Affects Cognition Hypothesis): Students’ perceived quality of the OBE-CDIO integrated education environment has a significant positive effect on their Cognitive Internalization Literacy.

*H2* (Environment Affects Practice Hypothesis): Students’ perceived quality of the OBE-CDIO integrated education environment has a significant positive effect on their Practical Externalization Literacy.

*H3* (Cognition Affects Practice Hypothesis): Students’ Cognitive Internalization Literacy has a significant positive effect on their Practical Externalization Literacy.

*H4* (Sequential Mediation Hypothesis): Cognitive Internalization Literacy mediates the relationship between Educational Environment Quality and Practical Externalization Literacy. That is, the educational environment indirectly promotes the development of Practical Externalization Literacy by enhancing Cognitive Internalization Literacy.

This model emphasizes that the primary and most direct effect of a high-quality integrated environment is to solidify students’ cognitive foundation (H1). This solid cognitive foundation (H3), combined with sustained environmental support (H2), jointly catalyzes excellent practical performance. H4 is key to this model, hypothesizing that the transformation from knowing to doing is the core mediating pathway through which the environment exerts its profound influence.

## 3 Research methods

### 3.1 Research design and participants

The study participants were undergraduate graduates from the 2019–2023 cohorts of the Chemical Engineering and Technology program at Nanjing Forestry University, a Double First-Class university. This major was chosen as a case study mainly due to its distinct feature of Forest-based Fine Chemicals, which embodies the resource-course-industry transformation path described in the Dual-Cycle model, and its documented extensive experience in implementing OBE-CDIO integrated education reform. An online questionnaire survey was administered via alumni networks. A total of 412 questionnaires were collected. After data cleaning (removing responses with excessively short completion times or obvious patterned answers), 366 valid samples were obtained (*N* = 366), yielding an effective response rate of 88.8%. The average age of the sample was 23.8 years (SD = 1.3), with males comprising 64.2%. All participants volunteered and participated anonymously.

Rationale for Retrospective Graduate Data: The use of retrospective self-reports from graduates is justified for several reasons in this context. First, engineering literacy, as a holistic assessment of competency attainment, is best evaluated at the culmination of the educational program when students have completed all integrated learning experiences. Second, graduates possess the necessary temporal distance to reflect on their overall perception of the educational environment and their developmental outcomes, potentially reducing transient state biases. Third, this approach aligns with the outcome-based philosophy of OBE, focusing on the enduring results of the educational process. While acknowledging the limitations of self-report and retrospective design, this method is deemed appropriate for capturing the perceived linkages between the educational environment and competence development from the learner’s perspective.

### 3.2 Measurement instruments

All core variables in this study were measured using a Likert 5-point scale (1 representing Completely Disagree, 5 representing Completely Agree). The OBE-CDIO Integrated Education Environment Quality Scale was self-developed, comprising 11 items across three dimensions: Curriculum Objective Alignment (X1, 4 items, e.g., The connection between course objectives and graduation requirements was very clear to the student, Cronbach’s *α* = 0.91), Practical Project Richness (X2, 4 items, e.g., Our course designs/experiments/projects possessed authentic engineering complexity and challenge, Cronbach’s *α* = 0.89), and Perception of Dual-Qualification Faculty Ratio (X3, 3 items, e.g., My professional teachers could effectively integrate theoretical knowledge with engineering practice cases, Cronbach’s *α* = 0.88).

The Engineering Literacy Attainment Scale was adapted based on the 12 graduation requirements of the Engineering Education Accreditation General Standard, resulting in 12 self-assessment items (Y1-Y12, see Table 1) to evaluate the enhancement level of various abilities after university cultivation. In this study, these were further divided into two subscales: the Cognitive Internalization Literacy subscale, comprising 5 items (Y1, Y2, Y4, Y5, Y12; Cronbach’s *α* = 0.92), and the Practical Externalization Literacy subscale, comprising 7 items (Y3, Y6, Y7, Y8, Y9, Y10, Y11; Cronbach’s *α* = 0.94).

**Table 1:** Graduation requirement attainment observation points Y1-Y12.

Observed variable	Observation point corresponding to the observed variable
Y1: engineering knowledge	Ability to apply mathematics, natural sciences, engineering fundamentals, and chemical engineering expertise to solve complex engineering problems in chemical engineering.
Y2: problem analysis	Ability to apply basic principles of mathematics, natural sciences, and engineering science to identify, formulate, and analyze complex engineering problems in chemical engineering through literature research, to obtain valid conclusions.
Y3: design/development of solutions	Ability to design solutions for complex engineering problems in chemical engineering, design systems, units, or processes meeting chemical product needs, and demonstrate innovation awareness while considering social, health, safety, legal, cultural, and environmental factors in the chemical engineering design process.
Y4: research	Ability to conduct research on complex engineering problems in chemical engineering based on scientific principles and methods, including designing experiments, analyzing and interpreting data, and synthesizing information to draw reasonable and effective conclusions.
Y5: use of modern tools	Ability to develop, select, and apply appropriate techniques, resources, modern engineering tools, and information technology tools for complex chemical engineering problems, including prediction and simulation of complex engineering problems, and to understand their limitations.
Y6: engineering and society	Ability to conduct reasonable analysis based on relevant background knowledge in chemical engineering, and to evaluate the impacts of professional engineering practices and solutions for complex engineering problems on society, health, safety, law, and culture based on the core socialist values of freedom, equality, justice, and the rule of law, and understand the responsibilities to be undertaken.
Y7: environment and sustainable development	Ability to understand and evaluate the impact of engineering practices for complex engineering problems in chemical engineering on environmental and social sustainable development, contributing to the goal of building a prosperous, strong, democratic, culturally advanced, harmonious, and beautiful modern socialist China.
Y8: professional ethics	Possess humanistic and social scientific literacy, social responsibility, understand and comply with engineering professional ethics and norms in chemical engineering practice, fulfill responsibilities, and actively practice the core socialist values of patriotism, dedication, integrity, and friendliness.
Y9: individual and teamwork	Ability to assume the roles of individual, team member, and leader in a multidisciplinary team when solving complex engineering problems in chemical engineering.
Y10: communication	Ability to use professional knowledge to effectively communicate and exchange information on complex engineering problems in chemical engineering design, planning management, and environmental consulting with industry peers and the public, including writing reports and design documents, giving presentations, and clearly expressing or responding to instructions. Possess broad international perspective and ability to communicate in cross-cultural contexts.
Y11: project management	Ability to understand and master the principles and economic decision-making methods of chemical engineering project management, and apply them in multidisciplinary environments.
Y12: lifelong learning	Possess awareness of autonomous and lifelong learning, understand development trends and future demands in chemical engineering, and have the ability to continuously learn and adapt to the development of chemical engineering technology.

### 3.3 Data analysis strategy

The data analysis employed quantitative methods, divided into three main parts: descriptive statistics, reliability/validity tests, and hypothesis testing. Firstly, SPSS 27.0 software was used for descriptive statistics, reliability analysis, and correlation analysis of the sample data to understand basic data characteristics and preliminary relationships among variables. Subsequently, AMOS 26.0 software was used for more in-depth Confirmatory Factor Analysis (CFA) and Structural Equation Modeling (SEM).

The specific analysis process followed these steps:

1. Exploratory Factor Analysis (EFA): Conducted on the 12 items of Engineering Literacy Attainment to preliminarily explore their latent structure and identify common factors explaining most of their variability. It was expected and aimed to verify that two factors would naturally emerge, corresponding to the theoretical constructs of Cognitive Internalization and Practical Externalization competence dimensions (Xu and Su, 2024).
2. Confirmatory Factor Analysis (CFA): Based on the EFA results, CFA was conducted to rigorously test the validity of the measurement models. This included verifying the two-factor structure of Engineering Literacy, and verifying the first-order three-factor structure and its second-order model for Educational Environment Quality, assessing the fit between theoretical models and observed data to ensure the accuracy and stability of core construct measurement (Shushanyan et al., 2025).
3. Structural Equation Model (SEM) Analysis (Guetterman and Manojlovich, 2024): Two competing models were constructed and compared. Model A (Direct Effect Model) specified that the three observed dimensions of environmental quality (Curriculum Objective Alignment X1, Practical Project Richness X2, Perception of Dual-Qualification Faculty Ratio X3) directly predicted a latent variable of Overall Engineering Literacy comprising all 12 graduation requirement items, used to test the direct effects of environmental elements on comprehensive competence (Simpfenderfer and Toutkoushian, 2024). Model B (Sequential Mediation Theoretical Model) embodied a more complex theoretical conception. It treated X1, X2, and X3 as indicators of the second-order latent variable Educational Environment Quality, explicitly distinguished engineering literacy into two first-order latent variables (Cognitive Internalization Literacy and Practical Externalization Literacy) (Kamár, 2024), and specified a sequential influence path of Educational Environment Quality → Cognitive Internalization Literacy → Practical Externalization Literacy. Model B aligns more closely with the Dual-Cycle model theoretical framework underlying this study, aiming to test the serialized mechanism from the macro empowering environment to individual cognitive internalization, and further driving explicit practical behavior (Gutiérrez-Aguilar et al., 2024).
4. Model Comparison, Mediation Testing, and Effect Calculation: To determine the best-fitting and most parsimonious model, we formally compared the nested Model A and Model B using a chi-square difference test (Δχ^2^). The model with the lower Akaike Information Criterion (AIC) and Bayesian Information Criterion (BIC) was considered superior. To test the significance of the hypothesized sequential mediation effect (H4), we employed a bias-corrected bootstrap procedure with 5,000 resamples. A mediation effect was considered statistically significant if the 95% bias-corrected confidence interval (CI) for the indirect effect did not include zero. Following the acceptance of the final model, we calculated and decomposed the standardized total effects (both direct and indirect) for each dimension of the educational environment quality construct on the outcome variable, practical externalization literacy.

## 4 Results

### 4.1 Measurement model: reliability and validity

Reliability, which can be referred to as consistency, aims to test the stability and consistency of a questionnaire. Specifically, it involves repeatedly measuring the same object using the same method, with the degree of consistency among the results. The most commonly used is Cronbach’s alpha reliability coefficient, which is an index measuring the consistency reliability among items. If items are independent measures of the same concept, they should have a certain degree of correlation. The closer Cronbach’s alpha is to 1, the higher the internal consistency reliability. Generally, the reliability coefficient for the total scale should ideally be above 0.8, between 0.6–0.8 is acceptable, and below 0.6 warrants reconsideration of the questionnaire design.

Reliability analysis indicated good internal consistency for all scales in this study. Validity was rigorously tested through both Exploratory Factor Analysis (EFA) and Confirmatory Factor Analysis (CFA). Detailed psychometric properties are summarized in Table 2.

**Table 2:** Summary of exploratory and confirmatory factor analysis results.

Construct and item	EFA loading (pattern matrix)	CFA loading (std. *β*)	S. E.	Critical ratio (C. R.)
Cognitive internalization				
Y1: engineering knowledge	0.82	0.79		
Y2: problem analysis	0.85	0.83	0.052	18.34
Y4: research	0.78	0.76	0.048	16.42
Y5: use of modern tools	0.75	0.71	0.049	14.96
Y12: lifelong learning	0.76	0.74	0.050	15.73
Practical externalization				
Y3: design/development	0.82	0.80		
Y6: engineering and society	0.71	0.68	0.057	13.71
Y7: environment and sustainability	0.73	0.70	0.058	14.23
Y8: professional ethics	0.76	0.74	0.054	15.46
Y9: individual and teamwork	0.79	0.77	0.053	16.53
Y10: Communication	0.83	0.81	0.049	17.89
Y11: project management	0.75	0.73	0.051	15.01
CFA model fit indices				
χ^2^/df	2.85	CFI	0.948	
TLI	0.940	RMSEA	0.071	

All factor loadings were significant at *p* < 0.001.

The EFA on the 12 engineering literacy items yielded a Kaiser-Meyer-Olkin (KMO) measure of 0.94, and Bartlett’s test of sphericity was significant (*p* < 0.001), confirming the data’s suitability for factor analysis. The analysis extracted two factors with eigenvalues greater than 1, cumulatively explaining 68.5% of the variance. Factor loadings aligned perfectly with theoretical expectations: Factor 1 (Cognitive Internalization) included items Y1, Y2, Y4, Y5, Y12 (loadings: 0.75–0.86); Factor 2 (Practical Externalization) included items Y3, Y6, Y7, Y8, Y9, Y10, Y11 (loadings: 0.71–0.83).

Subsequent CFA provided strong support for the proposed measurement models. The two-factor model for Engineering Literacy demonstrated excellent fit: χ^2^/df = 2.85, CFI = 0.948, TLI = 0.940, RMSEA = 0.071. The correlation between the two latent factors was *r* = 0.78, indicating approximately 61% shared variance and leaving 39% unique variance. This level of correlation is high but not indicative of redundancy, which would typically require *r* > 0.85 or shared variance exceeding 70%. More importantly, the distinction between cognitive internalization and practical externalization is theoretically and empirically justified on three grounds. First, differential prediction: prior research has shown that cognitive competencies (knowing) and practical competencies (doing) have different developmental trajectories and are predicted by different instructional approaches (Ryan et al., 2009). Second, differential outcomes: in engineering education specifically, cognitive mastery does not automatically translate into practical competence without explicit scaffolding (Kolb, 1984). Third, theoretical necessity: the mediation hypothesis (H4) specifically requires a two-factor structure because it posits that cognitive internalization mediates the environment-practice relationship; a unidimensional construct would collapse mediator and outcome, rendering the hypothesis untestable. The second-order model for Educational Environment Quality also showed an exceptional fit: χ^2^/df = 2.20, CFI = 0.970, TLI = 0.965, RMSEA = 0.057. All first-order factors loaded significantly onto the second-order construct (Objective Alignment = 0.88, Practical Richness = 0.82, Faculty Ratio Perception = 0.76, all *p* < 0.001). The overall Cronbach’s *α* for the full 11-item EEEQ scale was 0.93 (see Table 3). None of the subscale alphas (0.91 for Curriculum Objective Alignment, 0.89 for Practical Project Richness, 0.88 for Dual-Qualification Faculty Perception) exceeded the overall scale alpha, indicating that each dimension contributes uniquely without introducing redundancy. The overall Cronbach’s α for the full 12-item ELAS scale was 0.95 (see Table 3), with subscale alphas of 0.92 for Cognitive Internalization and 0.94 for Practical Externalization. Crucially, as shown in Table 3, all constructs demonstrated high Composite Reliability (CR > 0.88) and satisfactory Average Variance Extracted (AVE > 0.50), confirming convergent validity and scale reliability.

**Table 3:** Psychometric properties of measurement scales.

Construct and dimensions	Items	Cronbach’s *α*	KMO	AVE	CR	Example item/description
Educational environment quality	11	0.93	0.92	0.62	0.94	Second-order construct
Curriculum objective alignment (X1)	4	0.91	–	0.78	0.94	The connection between course objectives and graduation requirements was very clear to me.
Practical project richness (X2)	4	0.89	–	0.73	0.92	Our projects possessed authentic engineering complexity and challenge.
Dual-qualification faculty (X3)	3	0.88	–	0.71	0.88	My teachers effectively integrated theoretical knowledge with engineering practice.
Engineering literacy	12	0.95	0.94	–	–	Higher-order construct
Cognitive internalization	5	0.92	–	0.70	0.93	Items assessing engineering knowledge, problem analysis, research, modern tools, lifelong learning.
Practical externalization	7	0.94	–	0.68	0.95	Items assessing design, communication, teamwork, project management, ethics & society.

AVE, average variance extracted; CR, composite reliability.

To provide a consolidated overview of the factor structure, Table 2 presents the EFA pattern matrix and CFA factor loadings for all 12 measurement items.

### 4.2 Structural model analysis: direct and mediated effects

**Figure 2:**
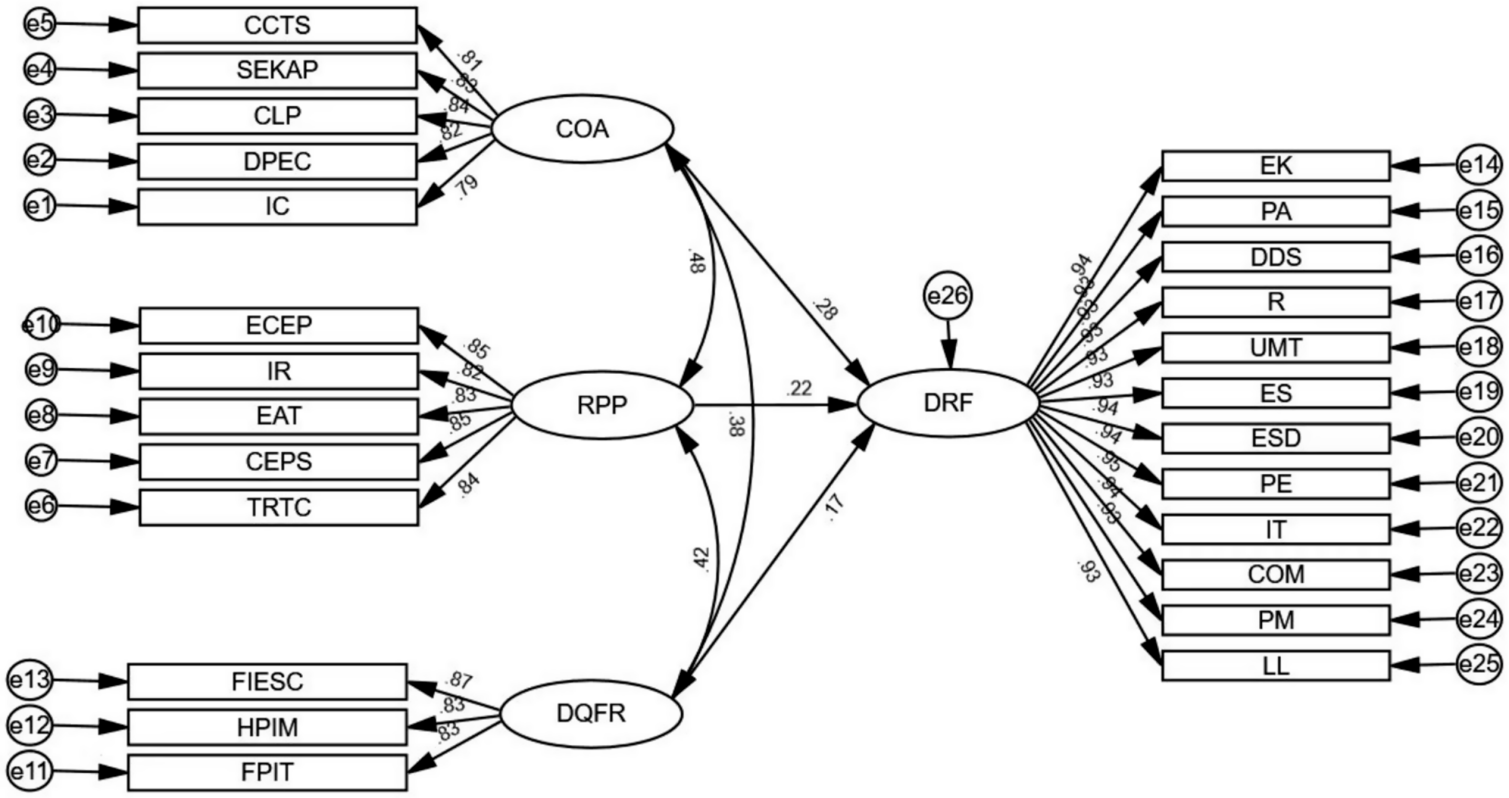
Direct effect model (Model A) with standardized path coefficients.

We first tested a direct effect model (Model A), as shown in Figure 2, in which the three observed environmental dimensions (X1, X2, X3) directly predicted a latent “Overall Engineering Literacy” variable. The model demonstrated a good fit to the data, which directly relates to the reliability of research conclusions. All three paths were significant, with Curriculum Objective Alignment (*β* = 0.282, *p* < 0.001) showing the strongest direct effect, followed by Practical Project Richness (*β* = 0.222, p < 0.001) and Perception of Dual-Qualification Faculty Ratio (*β* = 0.170, *p* = 0.002).

**Figure 3:**
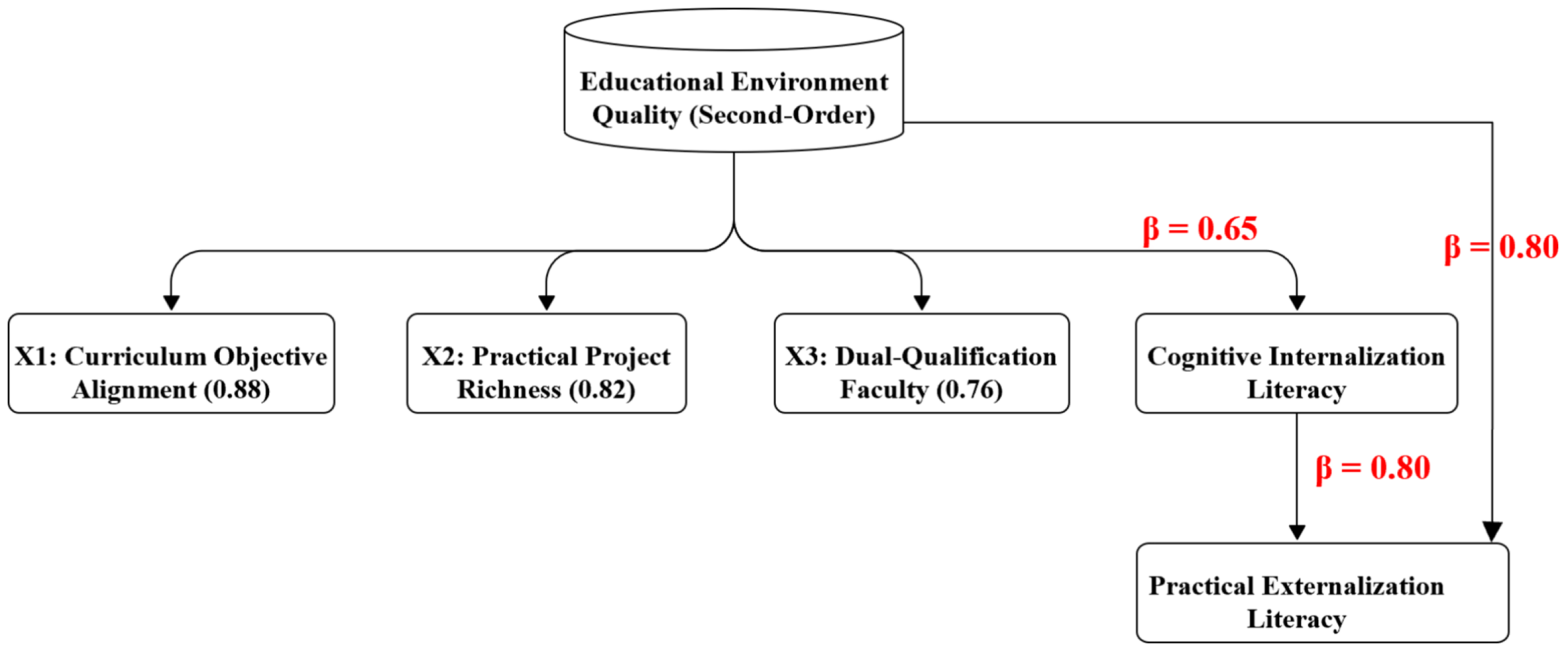
Final accepted structural model (Model B) with standardized path coefficients. *Second-order factor loadings are shown in parentheses. *** *p* < 0.001; n.s., non-significant (*β* = 0.08, *p* = 0.112, omitted in final model).

We then tested our theoretically hypothesized sequential mediation model (Model B), as shown in Figure 3. This model, which specifies “Educational Environment Quality → Cognitive Internalization Literacy → Practical Externalization Literacy,” showed an excellent fit that was slightly superior to Model A on key parsimony indices (RMSEA = 0.061, SRMR = 0.042). A formal chi-square difference test confirmed that Model B significantly outperformed Model A (Δχ^2^ = 29.0, Δdf = 6, *p* < 0.001). Information criteria also favored Model B, with lower Akaike Information Criterion (AIC) values (Model A = 567.4, Model B = 548.4) and comparable Bayesian Information Criterion (BIC) values (Model A = 781.9, Model B = 782.4), indicating superior fit with better parsimony (see Table 4). The standardized path coefficients revealed a strong and significant effect of Educational Environment Quality on Cognitive Internalization Literacy (*β* = 0.65, *p* < 0.001). In turn, Cognitive Internalization Literacy was a powerful predictor of Practical Externalization Literacy (*β* = 0.80, *p* < 0.001). A critical finding was that the direct path from Educational Environment Quality to Practical Externalization Literacy was non-significant (*β* = 0.08, *p* = 0.112) and was therefore omitted from the final model. This pattern of results provided initial support for H4, suggesting full mediation. The comparison of key statistics for both models is consolidated in Table 4.

**Table 4:** Model comparison and structural path analysis results.

Analysis aspect	Model A: direct effect model	Model B: sequential mediation model
Model fit indices		
χ^2^/df	2.541	2.380
CFI (comparative fit index)	0.963	0.960
TLI (tucker-lewis index)	0.959	0.955
RMSEA (root mean square error of approximation)	0.065	0.061
SRMR (standardized root mean square residual)	–	0.042
Model comparison		
χ^2^	457.4	428.4
df	180	174
Δχ^2^ (Δdf)	–	29.0 (6)***
AIC	567.4	548.4
BIC	781.9	782.4
Standardized path coefficients (*β*)		
X1 (objective) → outcome	0.282***	–
X2 (project) → outcome	0.222***	–
X3 (faculty) → outcome	0.170**	–
Env. Quality → Cog. Internalization	–	0.65***
Cog. Internalization → Prac. Externalization	–	0.80***
Env. Quality → Prac. Externalization (direct)	–	0.08 (n.s., removed)
Mediation and effect analysis		
Indirect effect (Bootstrapped 95% CI)	–	0.52*** [0.45, 0.59]
Effect size (Cohen’s f^2^)	X1: 0.085; X2: 0.053; X3: 0.031	Env → Cog: 0.73; Cog→Prac: 1.78
Hypotheses support	H2 partially supported (direct paths)	H1, H3, H4 fully supported; H2 not supported (direct path)

***p < 0.001, **p < 0.01, n.s. = not significant; CI, bias-corrected confidence interval based on 5,000 bootstrap samples; AIC, akaike information criterion; BIC, bayesian information criterion.

Based on the above indicators, the structural equation model constructed in this study performed well in terms of overall fit, parsimony, and explanatory power, effectively supporting subsequent path analysis.

### 4.3 Mediation effect test and total effect decomposition

To rigorously test the proposed mediation (H4), we employed the bootstrap procedure with 5,000 resamples. The indirect effect of Educational Environment Quality on Practical Externalization Literacy via Cognitive Internalization Literacy was 0.52 (calculated as 0.65 × 0.80). The 95% bias-corrected confidence interval for this indirect effect was [0.45, 0.59], which does not include zero. This provides strong statistical evidence for the significance of the mediation effect, fully supporting H4. Detailed bootstrap results for the mediation effect (H4) are presented in Table 5.

**Table 5:** Bootstrap results for the indirect effect (H4).

Indirect path	Estimate	Boot SE	95% BC CI	*z*	*p*	Bootstrap samples
Env. Quality → Cog. Internalization → Prac. Externalization	0.52	0.036	[0.45, 0.59]	14.44	<0.001	5,000

BC CI, bias-corrected confidence interval; Boot SE, bootstrapped standard error.

Within this established mediation framework, we decomposed the total standardized effects of each environmental dimension on the ultimate outcome, Practical Externalization Literacy. The total effect is the product of a dimension’s loading on the second-order Environment Quality factor and the two path coefficients in the mediation chain.

- Total Effect of Curriculum Objective Alignment = (Factor Loading 0.88) × (Path Coefficient 0.65) × (Path Coefficient 0.80) = 0.458.
- Total Effect of Practical Project Richness = (Factor Loading 0.82) × 0.65 × 0.80 = 0.426.
- Total Effect of Perception of Dual-Qualification Faculty Ratio = (Factor Loading 0.76) × 0.65 × 0.80 = 0.395.

These relative contributions are based on Model B (the final accepted sequential mediation model) and are expressed relative to the largest total effect (Curriculum Objective Alignment = 100%). In this framework, the relative contributions of Practical Project Richness and Dual-Qualification Faculty Ratio are 93 and 86%, respectively. This decomposition visually underscores the particularly robust foundational role of clear curriculum-goal alignment in the competence development chain, as its influence permeates through the cognitive internalization pathway most effectively.

## 5 Discussion

### 5.1 Core finding: revealing the sequential bridge between knowing and doing

The most significant theoretical contribution of this study lies in empirically demonstrating, through rigorous higher-order SEM analysis, a strong and sequential development path from Cognitive Internalization to Practical Externalization (*β* = 0.80) within the OBE-CDIO integrated education context. Environmental quality had no independent direct effect on practical externalization competency; its influence was primarily realized by solidifying the cognitive foundation. This finding profoundly reveals the internal logic of engineering competence development: Doing without deep understanding is water without a source, and knowing without practice orientation is a castle in the air. The success of the OBE-CDIO model precisely lies in ensuring the depth and direction of knowing through objectives (OBE’s contribution) and building the transformation bridge from knowing to doing through projects and faculty (CDIO’s contribution). Our model clearly shows that a central pillar of this bridge is the student’s internalized cognitive literacy.

### 5.2 Theoretical integration: linking the dual-cycle model with the Micro-level development path

This study achieves a macro–micro linkage in theoretical construction. First, the proposed Dual-Cycle Education Model depicts at the system level how OBE-CDIO integrated education shapes a high-quality, up-to-date empowering environment through the dynamic synergy of the Inner Cycle (objective calibration) and Outer Cycle (demand feedback). Building on this, this study further reveals the specific psychological mechanism through which this macro-environment functions at the individual level: the Empowering Environment → Cognitive Internalization → Practical Externalization path. This signifies the research’s progression from describing “how the educational system is designed” to explaining “how the educational environment influences student internal change.” Integrating dispersed environmental features into the higher-order construct Educational Environment Quality and connecting it with the deconstructed engineering literacy dimensions provides a more concise, powerful, and psychologically meaningful integrated explanatory framework.

### 5.3 Profound implications for engineering education practice

The results of this study offer three important implications for engineering education practice. First, the core status of OBE objectives must be upheld and deepened to solidify the foundation of the Inner Cycle within the Dual-Cycle Model. The data clearly show that the clear alignment of course objectives with graduation requirements is the first driving force propelling all subsequent learning processes. Institutions should invest resources in the refined design of cultivation plans, systematic mapping of curriculum maps, and ensure that program and course objectives are communicated clearly and effectively to teachers and students, thereby providing precise navigation for students’ cognitive internalization.

Second, the core positioning of CDIO projects needs to be re-evaluated, with focused efforts on bridging the transformation channel from knowing to doing. CDIO projects are not merely training grounds for skill development but also serve as touchstones and reinforcers for the outcomes of cognitive internalization. Project design should consciously guide students to mobilize their deeply understood knowledge (cognitive internalization) and apply it comprehensively, innovatively, and creatively in simulated authentic, complex, and uncertain engineering contexts (practical externalization), thereby completing the learning cycle from knowledge internalization to competency externalization. This requires educators, through the Outer Cycle mechanism of the Dual-Cycle Model, to continuously incorporate cutting-edge industry demands, ensuring the relevance and challenge level of practical projects.

Third, the deeper connotation of dual-qualification faculty development should be clarified, strengthening their supportive role in learning transformation. In the Chinese engineering education context, dual-qualification faculty refers to instructors who hold both academic degrees and industry-recognized practical qualifications, enabling them to bridge theoretical instruction and authentic engineering practice. The value of dual-qualification faculty extends far beyond transmitting practical experience; they can play the key role of cognitive coaches, helping students deeply connect specific engineering practical experiences with abstract theoretical knowledge, promoting the conditionalization and transfer of knowledge, thereby effectively reinforcing the transformation bridge from knowing to doing. They are indispensable key nodes connecting the Inner Cycle (systematic theoretical teaching) and the Outer Cycle (contextualized industrial practice).

### 5.4 Limitations and future research directions

This study employed a cross-sectional, retrospective self-report design, which carries several inherent limitations. First, retrospective reports are subject to recall bias: graduates may not accurately remember specific features of their educational environment, and positive or negative overall experiences may distort recall of specific dimensions. Second, halo effects may inflate correlations: graduates who feel successful in their careers may rate all aspects of their education positively regardless of actual environmental quality. Third, post-hoc rationalization may create artificial causal narratives where graduates unconsciously construct coherent stories linking past experiences to present outcomes. Most critically, the causal ordering implied by the model (environment → cognitive internalization → practical externalization) cannot be definitively established from cross-sectional data where all variables were measured at the same time point. Future research should employ longitudinal panel designs that measure students’ cognitive internalization at multiple time points (e.g., end of Year 2, Year 3, and post-graduation) and track how changes in environmental perceptions predict subsequent changes in competence, thereby providing stronger evidence for temporal precedence and causal direction. Furthermore, although common method bias may be a concern given the single-source, self-report nature of the data, the observed pattern of mixed significant and non-significant paths—consistent with theoretical predictions—and the strong discriminant validity of the measurement model suggest that such bias does not seriously threaten the validity of the conclusions (Podsakoff et al., 2003). Future research could further strengthen causal inferences by incorporating multi-source evaluations (e.g., peer or employer ratings) or temporally separated measurements.

Additionally, the current study operationalized students’ perceptions of environmental features (curriculum alignment, project richness, faculty qualification) as indicators of a functioning Dual-Cycle system. However, the dynamic, processual features of the Dual-Cycle model itself—such as the frequency of curriculum revision cycles, the intensity of industry feedback loops, or the mechanisms of stakeholder engagement—were not directly measured. Future research could develop instruments to capture these process variables and test whether they mediate the relationship between Dual-Cycle implementation and student-perceived environmental quality. Furthermore, the sample was drawn from a single program (Chemical Engineering and Technology with a Forest-based Fine Chemicals specialization) at a single Chinese Double First-Class university, which limits generalizability to other engineering disciplines, institutional types, or national contexts. Future research should replicate the model across diverse settings to establish boundary conditions and cross-cultural validity.

## 6 Conclusion

By constructing the macro framework of the Dual-Cycle OBE-CDIO Integrated Education Model and focusing on the micro-level development path of Empowering Environment → Cognitive Internalization → Practical Externalization, this study empirically reveals a clear logic for student competence development. The high-quality integrated education environment first promotes students’ deep understanding and internalization of engineering knowledge through a clear objective system (the core of the Inner Cycle). Subsequently, this solid cognitive foundation, under the guidance of high-level practical projects and dual-qualification faculty (empowered by the Outer Cycle), is efficiently transformed into explicit practical competencies for solving complex engineering problems and fulfilling social responsibilities.

The findings support the view that the OBE-CDIO model functions more as an integrated system than a simple superposition of elements, where macro-system design and micro-psychological mechanisms mutually support each other. It provides important paradigm reference and empirical evidence for engineering education shifting from emphasizing teaching to studying learning, and from focusing on overall effects to insighting into internal mechanisms. Future innovations in engineering education should focus more on optimizing the system operation of the Dual Cycles and the individual transformation chain of Environment-Cognition-Practice, thereby more systematically and efficiently cultivating outstanding engineers prepared for future challenges.

## Data Availability

The original contributions presented in the study are included in the article/supplementary material, further inquiries can be directed to the corresponding author.
